# Advances in computer-generated holography for targeted neuronal modulation

**DOI:** 10.1117/1.NPh.9.4.041409

**Published:** 2022-06-16

**Authors:** M. Hossein Eybposh, Vincent R. Curtis, Jose Rodríguez-Romaguera, Nicolas C. Pégard

**Affiliations:** a University of North Carolina at Chapel Hill, Department of Applied Physical Sciences, Chapel Hill, North Carolina, United States; b University of North Carolina at Chapel Hill, Department of Biomedical Engineering, Chapel Hill, North Carolina, United States; c University of North Carolina, Department of Psychiatry, Chapel Hill, North Carolina, United States; d University of North Carolina, Neuroscience Center, Chapel Hill, North Carolina, United States; e University of North Carolina, Carolina Institute for Developmental Disabilities, Chapel Hill, North Carolina, United States; f University of North Carolina, Carolina Stress Initiative, Chapel Hill, North Carolina, United States

**Keywords:** calcium imaging, optogenetics, computer-generated holography, sculpted light, neural modulation, photostimulation

## Abstract

Genetically encoded calcium indicators and optogenetics have revolutionized neuroscience by enabling the detection and modulation of neural activity with single-cell precision using light. To fully leverage the immense potential of these techniques, advanced optical instruments that can place a light on custom ensembles of neurons with a high level of spatial and temporal precision are required. Modern light sculpting techniques that have the capacity to shape a beam of light are preferred because they can precisely target multiple neurons simultaneously and modulate the activity of large ensembles of individual neurons at rates that match natural neuronal dynamics. The most versatile approach, computer-generated holography (CGH), relies on a computer-controlled light modulator placed in the path of a coherent laser beam to synthesize custom three-dimensional (3D) illumination patterns and illuminate neural ensembles on demand. Here, we review recent progress in the development and implementation of fast and spatiotemporally precise CGH techniques that sculpt light in 3D to optically interrogate neural circuit functions.

## 1 Introduction

Optogenetics has transformed experimental neurosciences with photosensitive molecular modulators that can activate^1^ or inhibit^2^ the activity of a population of neurons with light. A popular implementation of optogenetics relies on bulk illumination of a genetically defined population of neurons to modulate neural circuits. These techniques do not focus light precisely through brain tissue and cannot address individual neurons. They limit our ability to capture the wealth of information encoded within subpopulations of genetically identical but functionally distinct neurons. Since neurons located in close proximity to each other may serve very distinct roles in encoding brain functions,^3^ advanced light sculpting techniques that target large populations of individual neurons must sculpt light with spatial resolution that is on the order of the dimensions of the neuron’s soma, ∼20  μm in all three (x,y,z) directions^4^^–^^7^ to achieve single neuron specificity.

Since neural circuit functions typically involve the coordinated activity of millions of interconnected neurons, it is also necessary to address many individual neurons in parallel across large volumes of brain tissue. The primary technological challenge is that brain tissue is a dense heterogeneous medium where strong optical aberrations, optical scattering, and autofluorescence dramatically limit the resolution and depth at which individual neurons can be resolved. Hence, many light-sculpting technologies only target a small number of neurons simultaneously, severely limiting their ability to reliably modulate neural circuits.

Additionally, advanced light sculpting technologies must be able to modulate the activity of neurons at fast speeds matching the rapid dynamics of individual action potentials that often vary in terms of rate,^8^ timing,^9^ and synchronicity^10^ across neural circuits. Replicating these conditions using holography-based systems requires light sculpting capabilities with millisecond temporal resolution, in combination with neurons genetically engineered to express fast light-activated opsins with similarly short response times.^11^ Techniques that illuminate neurons sequentially by rapidly scanning a single high-resolution illumination spot from neuron to neuron^12^^,^^13^ lack the necessary speed and bandwidth to address neural populations at the rapid speeds of neuronal events. To fully enable the enormous potential of optogenetics, new light sculpting strategies must be developed where all three design constraints identified above must be addressed simultaneously.

The most promising approaches for parallel optogenetic modulation of neural ensembles are based on computer-generated holography (CGH).^4^^,^^5^^,^^7^^,^^14^^–^^17^ These advanced strategies decompose a laser light source into multiple focused beams that can simultaneously illuminate individual neurons. In combination with fast light-activated opsins,^11^ these scanless approaches can easily address large neural ensembles in parallel, with millisecond temporal precision and single-neuron resolution. To overcome the remaining experimental constraints for neural photostimulation, it is necessary to drive holographic light sculpting instruments with new, fast, and efficient CGH algorithms that move away from historical applications of CGH in three-dimensional (3D) display technology and are instead optimized to best perform optogenetic modulation tasks at the speed of neuronal events. Beyond optogenetic stimulation, progress in CGH algorithms and technologies similarly benefits neuronal imaging applications.^18^^–^^21^ Custom 3D illumination patterns can be used to selectively illuminate specific sections of tissue. The introduction of Holographic illumination in imaging systems reduces phototoxicity but also improves the signal-to-noise ratio of fluorescence measurements, as well as spatial resolution.

In the following review, we introduce the general principle of CGH along with an overview of the experimental and computational challenges associated with the technique. We then present several popular CGH algorithms and show how recently developed deep learning-based methods can achieve fast, and efficient holographic computations that directly benefits optogenetic applications. Last, we discuss the latest progress in advanced light sculpting techniques, with new technologies that circumvent the fundamental limitations imposed by coherent light in holographic systems. We show how new light sculpting techniques that modulate light both spatially and temporally can yield 3D illumination patterns with unprecedented spatial and temporal resolution and accuracy beyond the capabilities of conventional CGH systems.

## 2 Computer-Generated Holography

### 2.1 General Principles

**Fig. 1:**
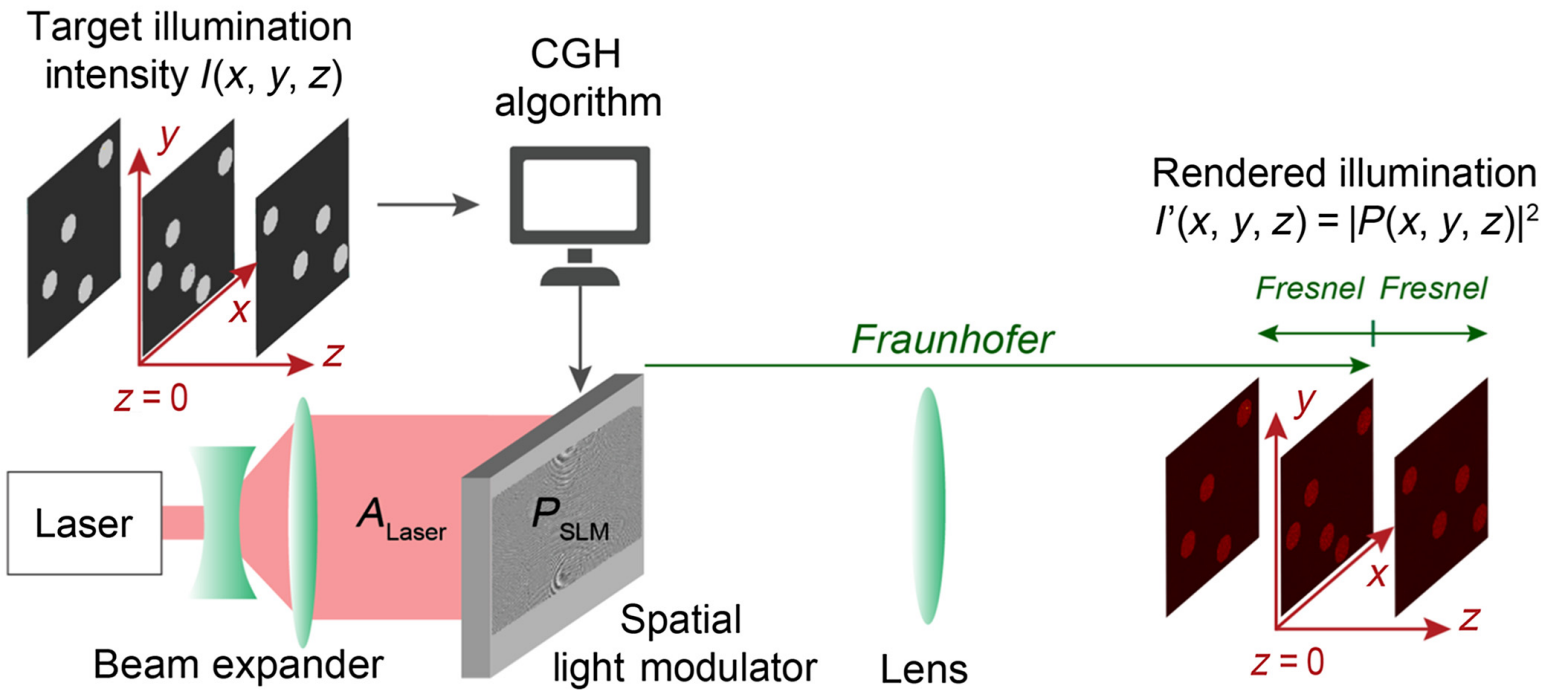
Example experimental configuration for CGH. A coherent light source with amplitude $A_{\mathrm{Laser}}(x,y)$, is modulated with an SLM. The shaped beam propagates through an optical system to redistribute light and render a 3D illumination pattern, $I^{\prime }$. The optical system configuration is termed “Fourier holography,” and places the modulator in the pupil plane, at a focal distance, f, from a convex lens. The complex field in the image plane, P(x,y,z=0) is determined by applying the Fraunhofer propagation equation [Eq. (2)] to the modulated beam at the SLM, then propagated to other depths, z using the Fresnel wave propagation equation [Eq. (3)]. The rendered illumination, $I^{\prime }$, is given by: $I^{\prime }={\left|P\right|}^{2}$. The CGH algorithm takes as input a target illumination pattern, I, in 2D or 3D, and aims to compute the SLM modulation parameters for which the rendered hologram, $I^{\prime }$, best matches I.

A popular optical system configuration for CGH is shown in Fig. 1. A computer-controlled optoelectronic device called spatial light modulator (SLM) is placed into the path of a coherent laser beam to engineer the wave and create a custom illumination pattern associated with the intensity distribution, I(x,y,z), through an optical system. In our example, the collimated laser beam propagates along the optical axis, z, illuminates the active surface of the SLM with a static amplitude profile, $A_{\text{Laser}}(x,y)$. The light modulator applies a custom modulation pattern, $M(x,y)=A_{\mathrm{SLM}}(x,y)e^{i\phi_{\mathrm{SLM}}(x,y)}$, either to the amplitude of the laser beam, $A_{\mathrm{SLM}}(x,y)$, or to its phase, $\phi_{\mathrm{SLM}}(x,y)$, or both. The engineered complex wave, $P_{\mathrm{SLM}}(x,y)$, directly after transmission (or reflection) exiting the modulator is given as

$$P_{\mathrm{SLM}}(x,y)=A_{\text{Laser}}(x,y)A_{\mathrm{SLM}}(x,y)e^{i\phi_{\mathrm{SLM}}(x,y)}.$$ (1)

The modulated wave, $P_{\mathrm{SLM}}(x,y)$, propagates through the optical system to render a 2D or 3D image, and defines the 3D complex field P(x,y,z). The light intensity distribution in the image space is then defined as $I^{\prime }(x,y,z)={\left|P(x,y,z)\right|}^{2}$. The task of a CGH algorithm is to identify the modulation pattern, M(x,y), for which $I^{\prime }(x,y,z)$ best matches the desired target intensity distribution I(x,y,z).

Figure 1 shows a popular configuration for CGH termed “Fourier holography” with a modulator placed in the pupil plane, at a focal distance f from a convex lens. In this configuration, the complex field at the center of the image plane at a distance, f, from the other side of the lens, P(x,y,z=0), is a 2D optical Fourier transform (FT) of the field at the modulator plane, $P_{\mathrm{SLM}}(x,y)$, and satisfies the Fraunhofer wave propagation model^22^

$$P(x,y,z=0)=\frac{1}{i\lambda f}\iint P_{M}(u,v)\exp[\frac{2i\pi (xu+yv)}{\lambda f}]du\;dv.$$ (2)

**Fig. 2:**
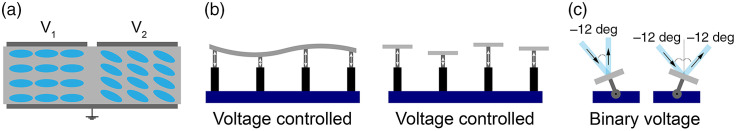
Common types of SLMs. (a) LC devices (LC-SLMs) consist of a 2D array of pixel-sized LCs. Their orientation modulates their birefringence and depends on the intensity of the electrical field across each pixel. (b) Continuous DMs consist of a flexible thin mirror that is mechanically deformed by electrical actuators. Segmented DMs eliminate cross-talk between actuators and enable a more precise pixelated modulation. (c) DMDs are MEMS made of miniaturize bistable mirrors. They are binary modulators as each micromirror can be electrically switched between two stable tilt angles.

The Fourier holography configuration is popular for its conceptual simplicity and gives the modulator access to redistribute light within the accessible (x,y) window in the image plane, at z=0. Wave computation can be rapidly estimated from the modulator plane to the image plane using fast FT (FFT) operations that are straightforward to implement, with computational complexity O(m*log(m)), where m is the number of pixels used to discretize the complex wave on the modulator. This modulation can be either applied to the phase or the amplitude depending on the type of modulator device (see Fig. 2).

The phase and amplitude of the coherent wave computed in the image plane using Eq. (2), fully determine the intensity distribution in the rest of the 3D volume. The volume of interest is decomposed into a series of parallel planes at predetermined depths (Fig. 1). The field at any location (x,y,z) is determined using the Fresnel wave propagation equation:^22^

$$P(x,y,z)=\iint \frac{P(u,v,0)}{\sqrt{i\lambda z}}\;\exp[\frac{i\pi ((x-u{)}^{2}+(y-v{)}^{2})}{\lambda z}]du\;dv.$$ (3)

Propagation to planes located before and after z=0 correspond to negative and positive values for z in Eq. (3), respectively. The modulator pixel size, $p_{s}$, the size (short axis length) of the SLM, h, the wavelength, λ, and the focal length of the lens, f, determine the vertical and horizontal span, $L=\lambda f/p_{s}$, of the accessible window along the (x,y) axes as well as the axial extent, $Z=\lambda f^{2}/(h*p_{s})$, of the accessible volume along the (z) axis. In practical applications, the span can also be adjusted by introducing an optical relay to scale holograms to the desired volume, for instance, by demagnifying the rendered hologram under the objective of a microscope. If the rendered hologram has dimensions L*L*Z in the image plane, and is demagnified by a magnification factor, $M_{X}$, then the reduced hologram dimensions are $\left(L/M_{X})*(L/M_{X})*(Z/M_{X}^{2}\right)$.

### 2.2 Light Modulation Devices

Figure 2 shows various types of light modulator technologies that are suitable for CGH. The most popular modulator technology for CGH is the liquid crystal SLM (LC-SLM), Fig. 2(a), which contains a 2D array of pixels that can be individually driven to modulate the phase (or the intensity with additional polarizing filters) of an incoming wave. LC-SLMs achieve continuous phase modulation by applying a voltage across each pixel, which temporarily modifies the birefringence of an LC. LC-SLMs exist both as transmissive (Fig. 1 shows a generic modulator in transmissive configuration) and reflective devices. The most popular technology is a reflective device known as LC on Silicon (LCoS). LCoS-SLMs are linear modulators, they have high reflectivity and high diffraction efficiency. They are usually used as phase modulators, with $M(x,y)=e^{i\phi_{\mathrm{SLM}}(x,y)}$, to preserve the amplitude of the incoming wave, and hence minimize the number of photons lost when the laser beam is reflected on the surface of the SLM. Continuous phase modulation is a preferred modulation strategy for CGH because the phase of a coherent wave in the Fourier domain carries significantly more information than its amplitude (please see the Supplemental Material for further explanation). Furthermore, LC-SLMs are key technologies for video-projectors and are commercially available at competitive costs. The main drawback of LC-SLM modulators is their speed, limited by the relaxation time of the nematic LC. The frame rate of LCoS-SLMs is typically under 400 frames per second (fps), with most LC-SLMs operating at standard video display rates (60 fps).

High-speed modulators such as deformable mirrors [DM, Fig. 2(b)] and digital micromirror devices (DMDs) [Fig. 2(c)] based on microelectromechanical systems (MEMS) technology are also commercially available and suitable for CGH applications. DMs [Fig. 2(b)] are reflective continuous phase modulators that operate at refresh rates of ∼10  kfps. DMs are popular in adaptive optical systems to rectify images distorted by rapidly fluctuating atmospheric aberrations in real-time. However, the implementation of DMs in CGH applications is rare, limited by the high cost and low pixel count of these devices. DMDs, [Fig. 2(c)], are 2D arrays of micromirrors that rapidly flip from one position to another with a binary instruction. Commercial DMD systems easily achieve refresh rates of up to 32,000 binary fps. However, DMDs unlike LC-SLMs are not continuous modulators and each pixel can only be digitally set to one of two states: on or off. Light reflected from pixels in the “off” state is typically discarded, which dramatically reduces power efficiency. Also, diffraction efficiency is low, and a substantial fraction of photons is lost to the zero order (or DC term) in the image plane. However, DMDs are inexpensive and mass produced for digital light projection display systems. They are typically implemented as binary amplitude modulators, rarely as binary phase modulators.^23^ Additionally, new MEMS technologies are currently being developed with innovative micromirror pixel designs that can be linearly actuated and even connected together for specialized applications of CGH such as fast refocusing of collimated laser beams.^24^

The choice of modulator technology affects the refresh rate, in fps, at which new modulation patterns can be placed on the device’s surface. For each frame, the modulation capability then depends on the type of pixel technology (Fig. 2). The most suitable modulator therefore depends on the CGH application, and whether the priority is speed (high fps), resolution (the number of pixels), rendering accuracy (lower with binary than with continuous modulation), and photon efficiency (reflectivity and diffraction efficiency). For simplicity, we will often discuss popular types of CGH systems that utilize phase-modulating SLMs. Nonetheless, the CGH principles we discuss can be easily generalized to other types of modulators.

### 2.3 Computational Challenges

The Fourier holography configuration (Fig. 1) includes a continuous phase modulating SLM, LC-SLM, in the pupil plane. SLMs in CGH systems are represented in forward models as operators performing a 2D modulation, M(x,y), that affect the amplitude or the phase (or both) of the laser beam transmitting or reflecting on the device’s surface. The aim of a CGH algorithm is to find a suitable phase modulation for the LC-SLM, $\phi_{\mathrm{SLM}}(x,y)$ so that the rendered 3D illumination pattern, $I^{\prime }(x,y,z)={\left|P(x,y,z)\right|}^{2}$, best matches the user-specified target illumination pattern, I(x,y,z).

The most common concern when implementing CGH in an optical system is that the user-specified target illumination pattern, I(x,y,z), may not be a feasible distribution of light. This is a common issue in optogenetic applications where target intensity distributions are constructed to match the physical location of neurons [e.g., Fig. 5(a)] without specifying how light is supposed to flow toward the targeted neurons and away from them. These target intensity distributions are clearly infeasible because they violate energy conservation principles along the optical axis, z

$$\forall \;z,\iint_{x,y}I(x,y,z)dx\;dy=I_{0}.$$ (4)

As a result, CGH is typically an ill-posed inverse problem for which an exact solution rarely exists. The expectation of the user is that the CGH algorithm should identify the pattern that must be applied to the modulator for which the feasible rendered pattern $I^{\prime }(x,y,z)$ best matches the desired target intensity distribution, I(x,y,z). Identifying the best approximation is a nonlinear, non-convex inverse problem, with as many variables as there are pixels on the modulator. Since it is practically impossible to exhaustively explore all the possible solutions, even the most advanced CGH algorithms offer no guarantees of identifying the best feasible solution.

### 2.4 Experimental and Physical Constraints

Both the optical system configuration and the choice of SLM technology impose limitations on the types of holograms that can be rendered. For instance, CGH systems may be by design limited to synthesizing illumination patterns by shaping either the phase or the amplitude of a coherent light source but not both. Their incomplete ability to modulate wavefronts will restrict the type and the quality of the holograms that can be rendered. The SLM’s pixel size, the number of available pixels, and the numerical aperture will determine the dimensions and the resolution of the illumination patterns that can be synthesized. Optoelectronics constraints, such as finite bit depth, pixel fill factor, pixel noise, nonuniformities of the SLM surface, and calibration drift will affect the diffraction efficiency, and the amount of light effectively placed in the desired illumination patterns. Together, hardware, electronic, and algorithm limitations result in a mismatch between the illumination pattern that the user requires and the pattern that is eventually rendered. This review article specifically discusses how tailored CGH algorithms and improved optical designs can circumvent some of these limitations to close the gap between the desired and rendered illumination patterns. Solving the inverse problem in CGH is currently an open problem, and evidence suggests that CGH algorithms are the main performance bottleneck.

## 3 CGH Algorithms

Existing CGH algorithms can be categorized into three groups based on the method of exploration: (1) iterative phase retrieval, (2) iterative optimization, and (3) noniterative deep learning techniques.

### 3.1 Iterative Phase Retrieval

**Fig. 3:**
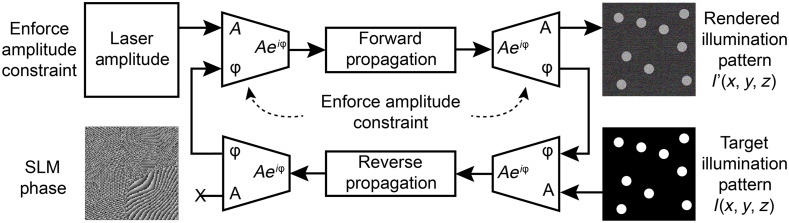
GS iterative algorithm for phase-only CGH. A randomly initialized complex field is propagated back and forth between the modulation plane and the image plane. At each step, phase information is retained but the amplitude is updated to either match the illumination profile of the laser in the SLM plane, and the desired intensity distribution, in the image plane. The algorithm typically converges to yield the desired phase modulation at the SLM plane.

The simplest strategy for CGH computation is the Gerchberg–Saxton (GS) algorithm.^25^ It is an exploratory method that digitally propagates a complex field back and forth between the image plane (in z=0), where the intensity distribution is rendered, and the SLM plane, where the wavefront is modulated while enforcing amplitude or phase constraints at each step. Figure 3 shows a typical implementation of the GS algorithm in Fourier holography systems (e.g., Fig. 1) with phase modulators, but the method seamlessly generalizes to other types of SLMs. The GS algorithm is popular^16^^,^^26^^–^^28^ because it is straightforward to implement, typically converges after a few iterations,^29^ and is easily extendable to 3D CGH.^30^^,^^31^ Many variations on the GS algorithm have been developed, yet all have the unique downside of requiring multiple iterations. The high temporal complexity of the algorithm inherently limits the computational speed, and the GS algorithm remains of limited usage in optogenetic excitation applications. Several strategies have been explored to address the issue of speed: compressed sensing methods^32^ have been demonstrated, and successfully reduce the computation time when the targeted volume is sparse, which is generally the case in neurostimulation applications. Other strategies rely on parallelizing computations with multicore central processing units and graphical processing units (GPU) with GPUs.^33^

Another common concern with GS algorithms is that while they often lead to visually recognizable solutions after only a few iterations, the solutions often have low fidelity to the target pattern. Increasing the number of iterations at the expense of longer computation times only marginally improves the quality of the solutions. The low fidelity of GS solutions can be attributed to the indirect optimization of the SLM parameters. The algorithm enforces constraints imposed by the target intensity distribution, I, which is generally unfeasible, but aims to converge to a feasible rendering, $I^{\prime }$. Looking for a feasible approximation by enforcing unfeasible constraints fundamentally limits the ability of the GS algorithm to retain the relevant information from each optimization cycle. The amplitude projection at each step discards parts of the feedback from the previous step, and the GS algorithm is unable to explore in detail the space of possible solutions in the vicinity of any potential high-fidelity solution.

### 3.2 Iterative Optimization

**Fig. 4:**
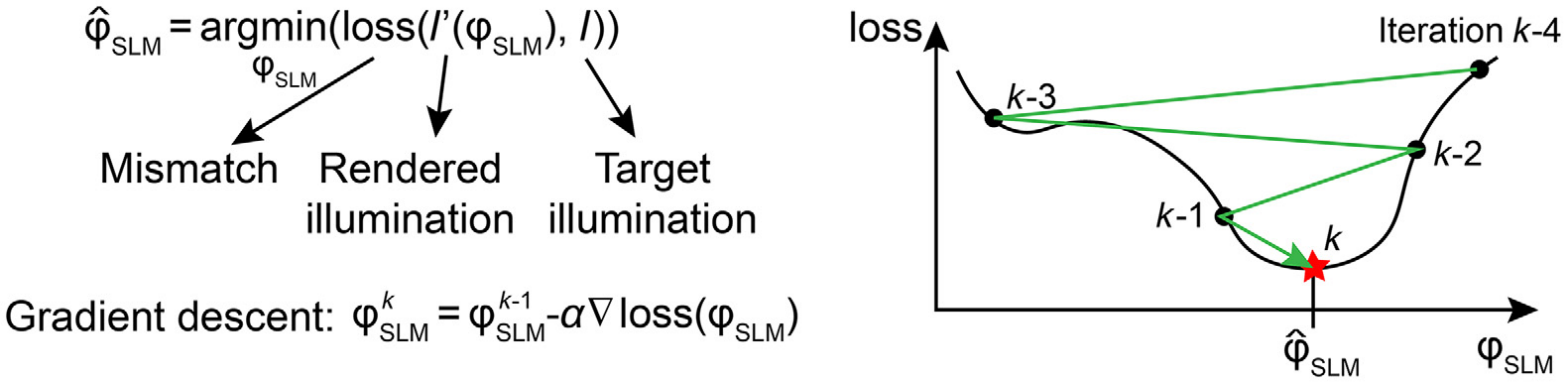
CGH algorithm with iterative nonconvex optimization using gradient descent. CGH computation is formulated as an optimization problem, with an explicit loss function measuring the mismatch between I, the desired hologram, and, $I^{\prime }$, the one obtained by applying the phase modulation ϕ on the SLM. The solution to the optimization problem, a phase modulation that minimizes the mismatch, is approximated using gradient descent optimization. Since the optimization problem is not convex, the algorithm may converge to a local minimum. In practice, this algorithm identifies better solutions than iterative GS methods, yet at the expense of further increasing the computation time.

CGH can be formulated as an optimization problem that can be solved by numerical gradient descent algorithms. The principle of optimization-based CGH is shown in Fig. 4. The illumination pattern, $I^{\prime }(x,y,z)$, rendered by a holographic setup (e.g., as in Fig. 1) is fully determined by the phase modulation $\phi_{\mathrm{SLM}}(x,y)$ applied to the SLMs while all other experimental parameters remain static. A forward model explicitly determines the rendered pattern, $I^{\prime }$, by simulating the propagation of the laser beam shaped by the phase modulation pattern $\phi_{\mathrm{SLM}}(x,y)$ through the optical system. The pixels on the SLM become the parameters that determine the rendered field, i.e., $I^{\prime }(x,y,z)=I^{\prime }(\phi_{\mathrm{SLM}})$. For a Fourier holography setup, as shown in Fig. 2, this operation reduces to the Fraunhofer propagation model to compute the complex field in z=0, followed by Fresnel propagation to compute it for other values of z. Both operations are differentiable with respect to the phase modulation $\phi_{\mathrm{SLM}}(x,y)$. The objective of an optimization-based CGH algorithm is to identify the modulation parameters $\phi_{\mathrm{SLM}}$ that minimize the mismatch between the target, I, and rendered, $I^{\prime }$, illumination patterns. The mismatch is quantified by an explicitly defined loss function that is also differentiable with respect to the phase modulation $\phi_{\mathrm{SLM}}(x,y)$. The resulting optimization problem is nonconvex, with as many dimensions as the number of pixels on the SLM. Approximate solutions to this problem can be identified using numerical techniques such as gradient descent,^34^ and methods based on Wirtinger derivatives that redefine CGH as a quadratic problem, which can be minimized with first-order optimization.^35^

**Fig. 5:**
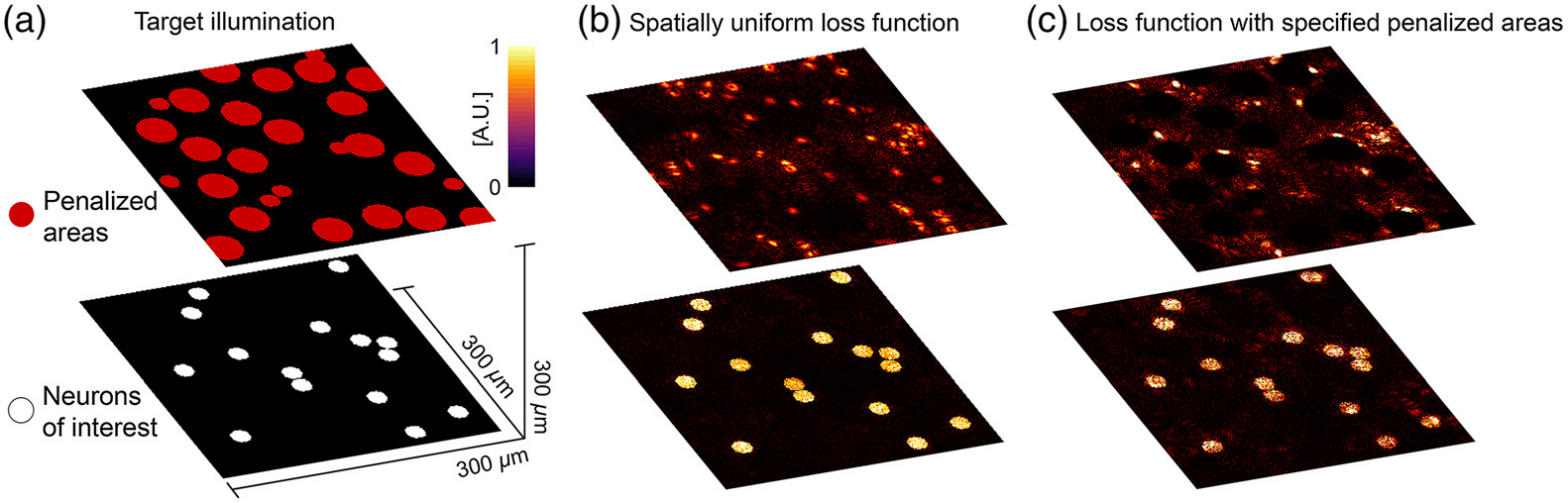
Customization of the loss function in CGH computation. (a) Hypothetical 3D distribution of a population of optogenetically encoded neurons. The objective is to stimulate a custom ensemble of neurons (labeled in white) while avoiding other neurons also expressing the opsin (labeled in orange). (b) CGH solution with a spatially uniform loss function. (c) CGH solution with a modified loss function that heavily penalizes the presence of light on nontargeted (orange) neurons.

One of the advantages of optimization-based CGH is that the explicit loss function can be tailored to steer the optimization toward holograms that best satisfy the desired outcome, rather than solely matching I(x,y,z) to $I^{\prime }(x,y,z)$. An illustrative example is shown in Fig. 5. We consider a hypothetical optogenetic 3D stimulation experiment where the objective is to stimulate a selected group of neurons within a larger population of neurons that are also expressing the opsin. The hologram we want to synthesize simultaneously places light in a group of neurons of interest while avoiding other neurons. Figure 5(a), shows in white, the targeted neurons that must receive light, and in orange, another cluster of neurons that are sensitive to light but not part of the targeted ensemble. The neurons to avoid are placed at another depth for illustrative purposes. First, we compute and render a hologram using the spatially uniform accuracy (AC) as loss function for CGH computation:

$$\mathrm{AC}(I,I^{\prime })=\frac{\underset{x,y,z}{\sum }(I.I^{\prime })}{\sqrt{\left[\underset{x,y,z}{\sum }I^{2}][\underset{x,y,z}{\sum }I^{\prime 2}\right]}}.$$ (5)

Simulation results, in Fig. 5(b) show that while the rendered hologram appears to be successfully optimized to simultaneously illuminate the neurons of interest with high visual contrast, the propagated field inadvertently illuminates many of the areas we attempt to avoid in the other plane. We then introduce a spatially weighted loss function by adding a penalty proportional to the amount of light intersecting the regions we intend to avoid. With the modified loss function, the CGH algorithm converges toward a task-optimized solution. The new rendered illumination pattern [Fig. 5(c)] now clearly avoids the areas labeled in orange while still depositing light in the targeted neurons. We note that the introduction of additional constraints comes at the expense of a slight loss of uniformity in the illumination on the targeted neurons. Nonetheless, this trade-off is beneficial in optogenetics applications where the ability to modulate neural activity primarily depends on the total amount of light received by each neuron.

Optimization-based methods typically converge toward solutions that are significantly better than those obtained with the GS algorithm. However, these techniques are also iterative and require gradients to be computed at every step, which further reduces computation speed. The step-size of the gradient descent optimization algorithm may be increased to accelerate the computation speed with fewer needed iterations or reduced to ensure convergence. Solutions that consistently have high-fidelity without the need to fine-tune parameters for each illumination pattern are preferred because they can deposit precise amounts of light and reliably activate or silence the individual neurons being targeted. Increased computation time severely limits the practicality of CGH algorithms in neuroscience research for optogenetic stimulation. To perform brain interfacing tasks, the neural ensembles that must be stimulated may depend on the most recently observed patterns of neural activity. Since a slow CGH algorithm cannot adequately leverage just-in-time information, the neurons that will be activated may no longer be relevant to the brain function being addressed by the time CGH computation is complete. Computing holograms beforehand is also not a suitable option since in a field of view with thousands of optogenetically accessible neurons, the number of possible combinations for addressable ensembles is prohibitively large. These considerations all highlight the importance of developing CGH algorithms that are reliable and consistent, both in the fidelity of the solutions, and in the computation time, to address neural ensembles with both the necessary spatial and temporal precision.

### 3.3 Noniterative Deep Learning Models

Deep learning models, specifically convolutional neural networks (CNN), are noniterative algorithms that rapidly compute nonlinear mappings. A CNN consists of hundreds of thousands of hierarchically structured trainable parameters. CNN computations are perfectly suited for an efficient implementation on GPUs where thousands of processing units can operate in parallel. The structure of CNNs enables high-throughput inference capabilities, therefore CNN models are suitable to solve inverse problems, and can infer solutions with fixed computational cost for individual inputs. Depending on the approach that is taken to train the parameters of a CNN, deep learning-based CGH algorithms are currently divided into two categories: supervised and unsupervised.

**Fig. 6:**
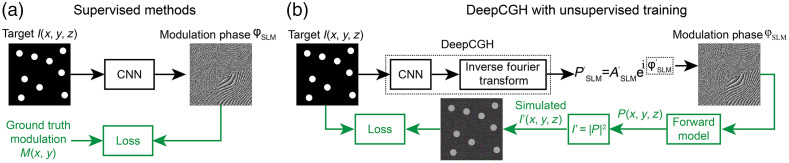
Deep learning-based CGH computation. (a) CNNs trained to take a target illumination pattern I(x,y,z) as input, can estimate, without iterations, a suitable modulation pattern for the SLM. The parameters of the CNN are optimized by comparing the output of the CNN with ground truth modulation patterns, calculated using another CGH technique or by direct simulation.^36^ The mismatch between estimated patterns M and ground truth is measured with a loss function. Supervised learning repeats the operation (green paths) on a large training dataset until the CNN accurately estimates holograms for training samples. (b) DeepCGH^37^ with unsupervised training. The hologram that results from the estimated modulation pattern is simulated with a forward model, and the mismatch between the simulated solution, $I^{\prime }(x,y,z)$ and the target, I(x,y,z), provides training feedback. This implementation of DeepCGH is shown for phase SLMs but the method naturally expands to other types of SLMs.

Figure 6(a) shows an example implementation of a CNN-based CGH algorithm^36^ with supervised training. The input of the CNN is the target illumination pattern, and the output is the estimated phase modulation pattern. In this example, we consider CGH with phase modulation, but the principles can again be easily extended to other types of SLMs. The parameters of the CNN must be optimized until the trained network can predict the desired modulation pattern that will yield high-fidelity renderings, $I^{\prime }$, when the model receives previously unseen distributions, I, as input. During the supervised training process, the CNN will “learn” from a large set of pairs of target illumination patterns, which are inputs to the CNN, and their corresponding ground truth phase modulation, i.e., the expected output of a hypothetical perfectly trained CNN presented with the associated input distribution. The ground truth phase modulation data must be determined using another CGH algorithm^38^^,^^39^ or simulated.^36^^,^^38^ During training, the mismatch between the predictions of the CNN and the ground truth phase modulation is quantified using a loss function (e.g., mean squared error). The parameters of the CNN are updated with gradient descent optimization to minimize the loss function for all the samples in the training dataset.^36^^,^^37^^,^^40^ The supervised training process iterates through the entire training dataset several times (repetitions are termed epochs) and is generally a time-consuming process. However, once the CNN is trained, it is able to calculate the phase modulation that will best render previously unseen input patterns with a single pass of the input data through the CNN, with fixed computational complexity. This operation, termed inference no longer requires any iterations. The implementation of CNN models dramatically reduces the computation time of CGH algorithms, at the expense of a complex training operation that must only be performed once.

An impediment of deep learning-based CGH methods with supervised training is the training dataset sets the limit for performance. The CNN does not learn to identify the best possible holograms, but to identify the CGH solution that would be found with the CGH algorithm used to generate the training dataset. In other words, a supervised CNN learns to mimic another CGH algorithm and is highly unlikely to outperform it. A possible option to circumvent this issue is to perform supervised learning with a clearly feasible training data set that is generated backward. Random SLM modulation patterns are matched to their rendered illumination pattern reclassified as target intensity distributions.^36^ While this approach addresses the aforementioned problem, it has the downside of restricting the training data that will be presented as input to the CNN to random and feasible target illumination patterns. As the patterns employed during this operation are generally not feasible (see Sec. 2.3, users instead input a distribution for which they seek a well-matched feasible approximation. The training dataset is therefore not representative of the data that will be used during subsequent CNN operations, and the CNN is unlikely to learn how to handle infeasible inputs.

### 3.4 DeepCGH: A CGH Algorithm with Unsupervised Training

DeepCGH with unsupervised training addresses the drawbacks of supervised training CGH techniques. The algorithm’s structure and training are shown in Fig. 6(b). Unsupervised training is achieved by simulating the rendered illumination pattern, $I^{\prime }(x,y,z)$ from the CNN-estimated phase modulation pattern (green path) and directly comparing it to the target illumination pattern I(x,y,z). Gradient descent optimization is used to optimize the CNN parameters to minimize the mismatch between $I^{\prime }(x,y,z)$ and I(x,y,z). This training strategy only requires a large dataset of representative target illumination patterns I(x,y,z), but the ground truth modulation patterns are not needed. For both feasible and unfeasible illumination patterns, the CNN in DeepCGH approximates a feasible illumination pattern that best matches the input pattern. Due to unsupervised nature of the training that eliminates ground truth data, DeepCGH is not limited by the performance of another CGH algorithm. A brief introduction and tutorial on the DeepCGH code are available in the Supplemental Material.

### 3.5 Comparison of Different CGH Algorithms

**Fig. 7:**
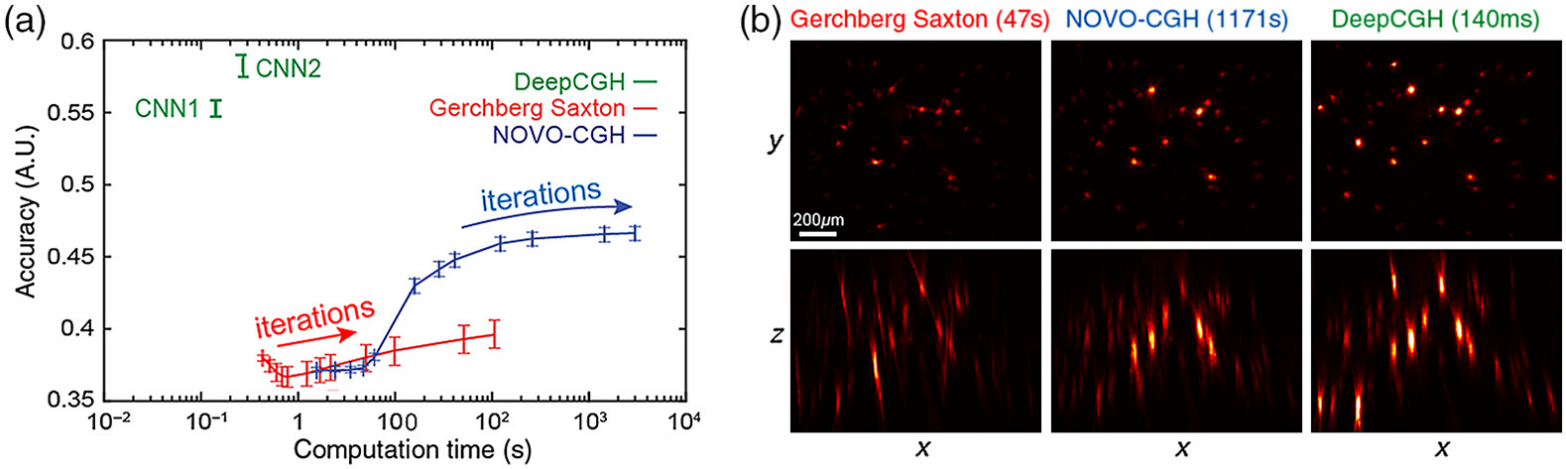
(a) Accuracy, a measure of mismatch between I(x,y,z) and $I^{\prime }(x,y,z)$, of 1000 random rendered holograms is shown as a function of the computation time for iterative CGH techniques and DeepCGH. DeepCGH solutions have significantly higher accuracy with computation time that is orders of magnitude faster than iterative techniques. CNN1 and CNN2 compare two distinct CNN model sizes and show that increasing the model size can improve the accuracy of renderings, though at the expense of extended computation time. (b) Experimental results in a two-photon holographic microscope compare the two-photon absorption induced in a fluorescent calibration slide with holograms of identical target distribution computed with different CGH algorithms and the computation time for each CGH solution. All three holograms are recorded with the same amount of laser intensity intercepting the slide.

Figure 7(a) compares the accuracy, a measure of mismatch (see Supplemental Material) of CGH solutions obtained with DeepCGH and iterative techniques^25^^,^^34^ as a function of their respective computation time. In these examples, the loss function for DeepCGH and NOVO-CGH is accuracy. The figure shows the averaged computation time for 1000 previously unseen 3D distributions with 11 planes and a resolution of 1024×1024  pixels. As can be seen in Fig. 7(a), obtaining a satisfactory CGH solution with the GS algorithm is not guaranteed, even after many iterations and extended computation times. Optimization-based methods such as NOVO-CGH offer significantly better CGH solutions than GS. However, greater accuracy comes at the cost of further increasing computation time. Optimization-based CGH algorithms are likely to require fine-tuning of the parameters, such as the step size, for each individual target illumination pattern. This will greatly reduce the efficiency and practicality of these methods. Fine-tuning of the algorithm is not needed with deep learning-based models as long as the data at the input of the CNN is normalized. The only remaining downside of deep learning-based CGH is that a new model must be trained anytime the CGH forward model is modified. Changing the number or the location of the depth planes that discretize the addressable volume or the resolution of the holograms requires a new model to be trained.

DeepCGH operates in fixed time and is orders of magnitude faster than iterative techniques. Also, unsupervised training allows DeepCGH to identify solutions with significantly higher fidelity compared with iterative methods, which is highly valuable in holographic optogenetic applications where off-target light may stimulate nontargeted neurons. The benefits of improved accuracy are highly valuable in two-photon holographic photostimulation systems where misplaced light contributes to unwanted tissue heating. To demonstrate these benefits, we consider a 3D target intensity pattern, I(x,y,z), that consists of randomly located disks at five depth planes representing a 3D intensity distribution that would be used to simultaneously illuminate randomly distributed neural targets with two-photon excitation.

To experimentally compare the holograms obtained with iterative CGH techniques^25^^,^^34^ and DeepCGH,^37^ we compute three holograms that correspond to the same target intensity distribution, I, but independently with the three algorithms being compared. We then experimentally rendered the holograms in a microscope customized for multiphoton holographic excitation. The experimental setup is similar to the Fourier configuration shown in Fig. 1, with a high-power femtosecond laser light source, and an additional tube lens and microscope objective to demagnify the 3D hologram. We measured the 3D distribution of fluorescence induced by two-photon absorption using a calibration slide that is mechanically scanned throughout the volume of interest while 2D images of the uniformly fluorescent thin film at the surface are recorded with a substage camera. Quantitative measurements were acquired for each method by displaying the three SLM patterns and capturing the corresponding fluorescence images, with the same amount of laser power. The acquisition sequence was randomized to compensate for potential photobleaching in the calibration slide. Figure 7(b) shows 3D reconstruction of fluorescence induced by two-photon excitation in the volume of interest. Experimental results suggest that by adopting DeepCGH to control multiphoton holographic microscopes, improvements in hologram accuracy enhance the amount of two-photon excitation in neural targets, which improves optogenetic stimulation without requiring additional optical power. Considering that current multiphoton systems are limited in their performance by the amount of heat that the infrared light deposits in the brain tissue,^41^^,^^42^ high-performance CGH algorithms such as DeepCGH represent inexpensive software solutions to increase the number of neurons that can be optically targeted in parallel.

CNN-based models can be customized to specific tasks by selecting representative training datasets, and by tailoring the loss function during training. This is relatively easier for DeepCGH with unsupervised training because the ground truth CGH solutions are not explicitly provided.^37^^,^^43^ The user only needs to provide the specialized dataset to train the model. The adjustable capacity of the CNN model for DeepCGH also introduces a trade-off between hologram fidelity and computation time. This flexibility can be leveraged to configure DeepCGH to specific tasks or to match the modulation hardware’s available refresh rate and optimize the usage of computational resources. Figure 7(a) shows an example trade-off with two different DeepCGH models, labeled CNN1 and CNN2. The capacity of the two CNNs is different but the dimensions of the CNN’s input and output remain identical. The CNN2 model has 50% more parameters than CNN1 and yields solutions with higher fidelity, at the cost of slightly more computational time.

Deep learning-based CGH methods are gaining popularity beyond applications in neurosciences^38^^,^^44^ and are on track to successfully address the computational challenges highlighted in Sec. 2.3 in the near future. The next performance bottleneck for light sculpting with CGH techniques is currently imposed by the experimental hardware, and addressing it will require the development of new light modulation technology.

## 4 Time-Multiplexed Light Sculpting Techniques

Conventional CGH techniques can only shape light in 2D. As shown in Sec. 2, the 2D phase and amplitude at the plane of the modulator [see Eq. (1)] fully determine how light will propagate throughout the rest of the 3D volume. Therefore, the 3D illumination patterns that can be rendered by engineering a coherent light source are only an extremely small subset of all the feasible 3D illumination patterns that we would want to be able to synthesize. Another consequence of this dimensional discrepancy, is that custom, user-defined 3D target illumination patterns are statistically extremely unlikely to be feasible. A clear indicator that coherent CGH only offers dramatically limited light sculpting capabilities is that holograms routinely exhibit speckle noise. Speckle noise is a high-frequency perturbation pattern that appears as a result of uncontrolled interferences as the engineered coherent waves propagate throughout the entire volume including where the 3D image is rendered. Speckle is a clear indicator of wave coherence, easily noticeable in most Laser light sources, and an unwanted artifact for optogenetic applications,^45^ even if small amounts of speckle may be tolerated as long as most of the light is adequately focused anywhere on the neuron’s soma. Nonetheless, tissue aberrations and scattering amplify the effects of speckle perturbations and rapidly degrade hologram quality. Eventually, neurons located in deeper layers of brain tissue cannot be addressed individually as the amount of perturbations drastically increases.

To enable volumetric light sculpting with reduced speckle and beyond the capabilities of traditional CGH systems, it is necessary to consider new light sculpting hardware and algorithms. Specifically, new light modulation strategies must be developed that are not restricted by the constraints imposed by wave coherence. Eliminating speckle noise in CGH-synthesized illumination patterns is a well-known strategy to circumvent the constraints imposed by coherent wave properties. Despeckling techniques are popular in holographic 3D displays and can be easily achieved by introducing fast, random temporal fluctuations in the laser light source, for instance with a rotating diffuser.^46^^,^^47^ In all these applications, the despeckling is perceived as long as the integration time of the human eye, remains significantly slower than the random fluctuations applied to the laser light source. At faster timescales, the effect of wave coherence would still be perceivable. This type of illumination is typically referred to as partially coherent light.

Also similar to the human eye, the response kinetics of bacterial opsins typically range from one to hundreds of milliseconds depending on the opsin type, far slower than the refresh rate of high-speed modulators. Therefore, it is theoretically possible to synthesize perceptually incoherent patterns of light, as long as their individual coherent components are refreshed at faster rates than the opsin’s response time. From the perspective of the opsin, the effective optical stimulation pattern will be the total amount of received photons, averaged across the integration time of the opsin. Introducing fast modulation capabilities as an extra degree of freedom for light sculpting dramatically increases the number of degrees of freedom for light sculpting. Yet, LC-SLMs [Fig. 2(a)] that are popular in CGH systems are too slow to modulate light at submillisecond scales because their frame rate is limited by the relaxation time of LCs. Recently, modulation speeds were obtained^48^ by dividing an LC-SLM in tiles, sequentially illuminated with a mechanically scanned laser beam, but this approach adds complexity to the device and introduces a trade-off between modulation speed and spatial resolution. An alternate strategy is to leverage the benefits of other, faster types SLMs, [Figs. 2(b) and 3(c)], which can refresh 2D modulation patterns on their surface at multi-kilohertz frame rates. These technologies have the ability to sculpt light with tens, or even hundreds of sequentially displayed frames that can be sequentially displayed well within the response time of opsins.

### 4.1 3D Multisite Random Access Photostimulation

**Fig. 8:**
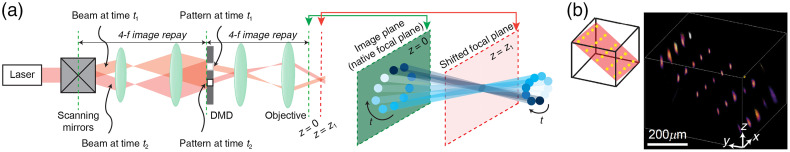
(a) Optical configuration of 3D-MAP. A collimated Laser beam is projected to the surface of a digital micromirror device to be shaped spatially while a pair of galvomirrors synchronously controls the illumination direction of the incoming wave. (b) Example 3D illumination distribution obtained with 10, rapidly superimposed frames with a revolving oblique illumination.

Recently, a new light sculpting technique termed 3D multisite random access photostimulation (3D-MAP)^49^ has been developed to synthesize partially coherent illumination patterns with enhanced 3D focusing capabilities (Fig. 8). 3D-MAP relies on a pair of galvomirrors to illuminate the DMD from custom angular directions, whereas the DMD selectively opens binary windows to modulate light spatially in the image plane. The galvomirrors and the DMD are synchronized to illuminate neurons with a fast sequence of beams of light projected onto them but originating from a broad range of high-incidence illumination directions. From the perspective of a much slower opsin, 3D-MAP provides independent control of light sculpting capabilities, both in the spatial, and in the angular domain, which enables remote focusing with enhanced depth specificity. The illumination pattern rendered by each frame placed on the DMD remains constrained by the coherence properties of the laser light source, but the time-averaged illumination perceived by neurons when frames are displayed in a rapid sequence is a 3D illumination pattern that precisely address targeted neurons. The resulting time-averaged illumination distribution is not limited by constraints imposed by wave coherence, and could not have been synthesized by a traditional CGH system with a static modulation pattern.

### 4.2 Time-Multiplexed Computer Generated Holography

3D-MAP has been successfully implemented to rapidly map synaptic connectivity in upper layers of brain tissue and is easily scalable to address many neurons in parallel. However, its operation relies on a bulky microscope and mechanical mirrors, which are subject to misalignment errors. To address these limitations, a time-multiplexed CGH technique termed Dynamic CGH^50^ synthesizes 3D illumination patterns by rapidly displaying a sequence of jointly optimized binary modulation patterns on the surface of a DMD^51^^–^^53^ placed in a Fourier CGH configuration.

**Fig. 9:**
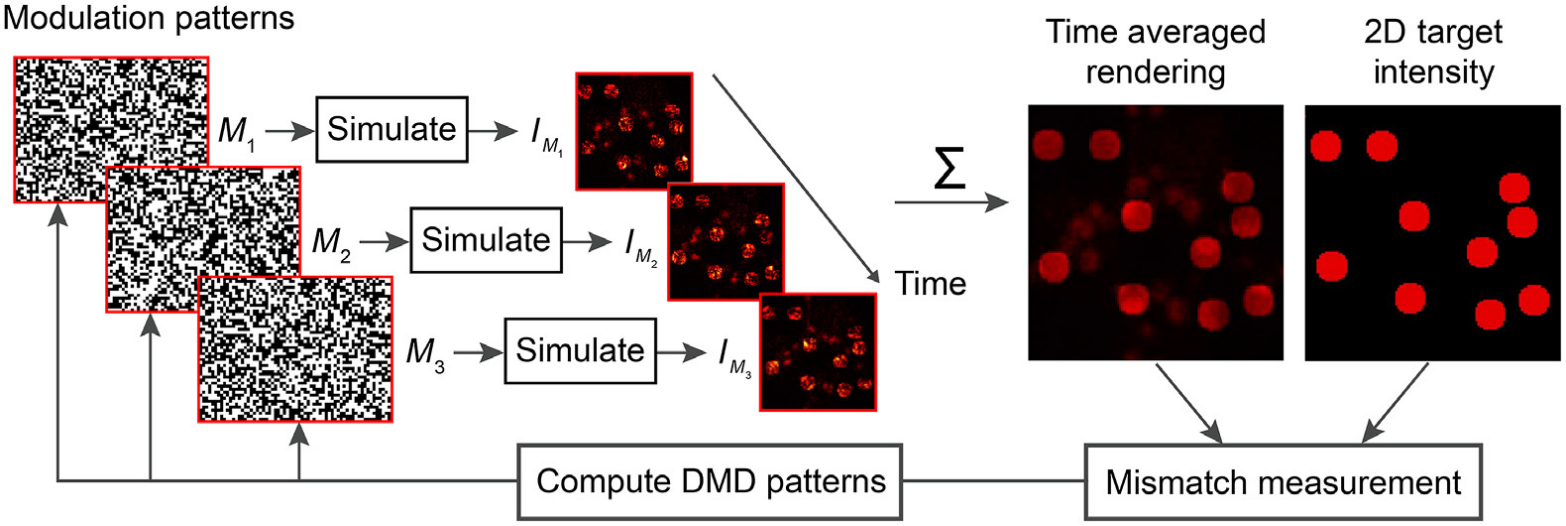
Dynamic CGH relies on an algorithm to jointly optimize a set of modulation patterns $M_{1}(x,y)\ldots M_{n}(x,y)$ so that the renderings resulting from these patterns, $I_{M_{1}}(x,y)\ldots I_{M_{n}}(x,y)$, accumulate to a time-averaged rendered illumination that best matches the user-specified target illumination distribution. The co-optimized modulation patterns are rapidly displayed on a high-speed SLM (e.g., digital micromirror device). As long as the receptor has a significantly slower response to light, the distribution it perceives corresponds to the time-averaged sum of the coherent holograms successively rendered in the sequence.

The principle of dynamic CGH is shown in Fig. 9. An algorithm computes a series of binary amplitude modulation frames, $M_{1}(x,y)\ldots M_{n}(x,y)$, (n=3 in Fig. 9) that are simultaneously optimized so that the aggregated contributions of their corresponding renderings, $I^{\prime }(x,y,z)=\underset{i}{\sum }I_{M_{i}}^{\prime }(x,y,z)$, best matches the target illumination pattern, I(x,y,z). Experimentally, the frames will be rapidly displayed on a DMD at speeds that are orders of magnitude faster than the response time of the optical receptor. From the perspective of an optogenetically encoded neuron with a slower response time, the perceived illumination pattern corresponds to the aggregated contributions of each individual illumination pattern in the sequence. The number of frames available for 3D light sculpting depends both on the refresh rate of the DMD and the characteristic response time of the stimulation target. For example, given a DMD with a refresh-rate of 12,000 KHz and a relatively high-speed ChroME-based opsin with a response time of 25 ms,^11^ up to 300 frames can be used in a single sequence to render any time-averaged distribution, and 3D time-averaged illumination patterns can be refreshed up to 40 times per second.

Since DMD pixels can only be switched between two states (on or off), the space of feasible coherent holograms for a single, binary DMD frame is further restricted than with continuous SLMs. However, as with 3D-MAP, multiplying the number of available frames for hologram synthesis dramatically increases the number of available degrees of freedom available to the algorithm to identify a suitable decomposition. As a result, dynamic CGH can render high-fidelity incoherent distributions of light that are not feasible with coherent, static CGH techniques while ensuring that individual frames remain feasible despite limited wave modulation capabilities at the surface of the DMD. Dynamic CGH can be implemented by modifying existing CGH algorithms to jointly optimize the dynamic CGH frames. The frames can be optimized sequentially using a modified GS algorithm^50^ simultaneously using gradient descent optimization, or deep learning-based models such as DeepCGH.^53^ A higher number of jointly optimized frames enables higher-fidelity time-averaged results but at the price of increased computational cost. Therefore, deep learning-based models are great candidates for these applications as they leverage parallel processing and can process multiple frames concurrently.

**Fig. 10:**
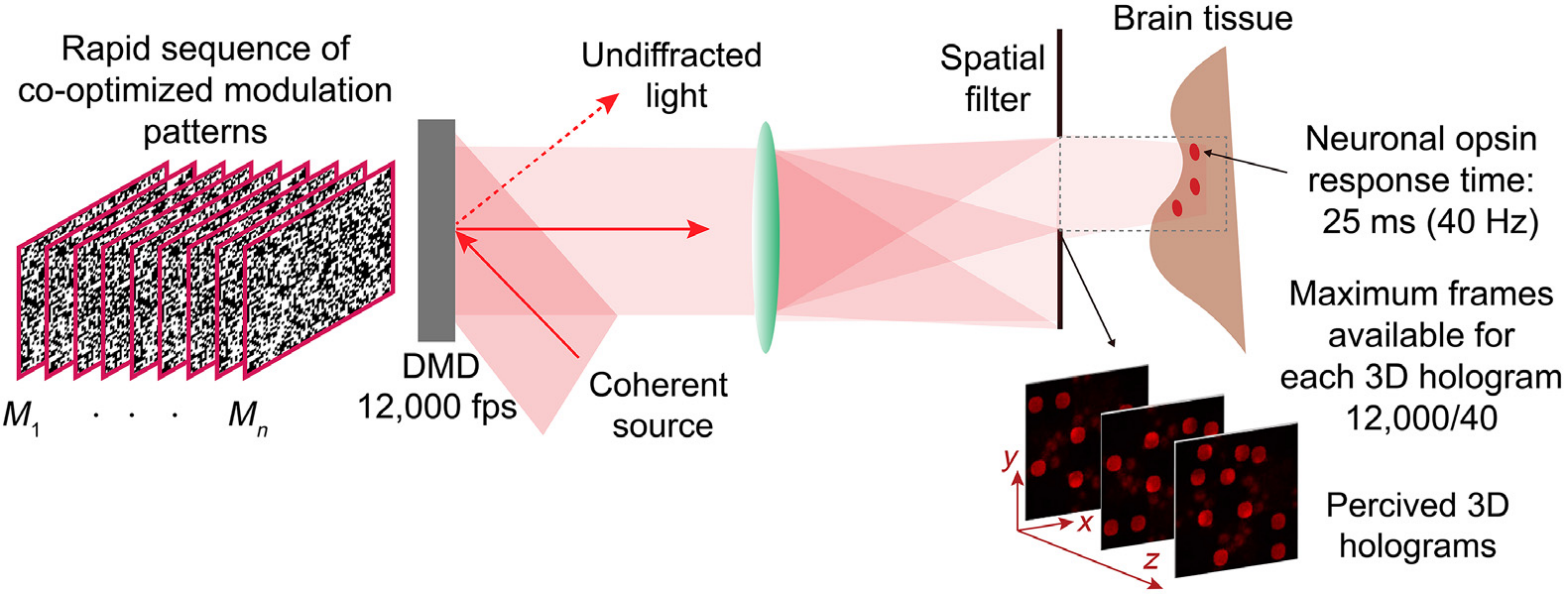
Dynamic CGH can be implemented with inexpensive hardware in a compact format. In the proposed configuration, the DMD modulates a collimated laser beam with a sequence of binary amplitude patterns that are computed by the Dynamic CGH algorithm. A spatial filter eliminates undiffracted light, secondary diffracted orders, and symmetrical copies of the rendered image. The holograms are synthesized in the remaining accessible window and can be used to render images in a human eye, or to stimulate 3D neural circuits in the brain with optogenetics, by taking advantage of the response speed of opsins, which is far slower than the refresh rate of the DMD.

The additional flexibility afforded by multiframe decomposition allows dynamic CGH to be highly adaptable to a variety of experimental conditions. In Fig. 10, we show how dynamic CGH can be implemented with compact, off-the-shelf hardware.^54^ An inexpensive DMD modulates a collimated laser beam by applying binary amplitude patterns, computed with our dynamic CGH algorithm. In the Fourier holography configuration, a spatial filter is required to eliminate undiffracted light, secondary diffracted orders, and symmetrical copies of the rendered field. The remaining opening defines the accessible window for hologram synthesis.

## 5 Conclusion and Perspectives

One of the greatest ongoing endeavors of systems neuroscience is to decipher how the brain integrates information within neural circuits to allow processes such as perception, cognition, and behavior to occur. To study such complex phenomena, neuroscientists require tools to read and write the activity of the brain with the spatial resolution of individual neurons, and at the temporal resolution of individual action potentials. These neural interfaces must also operate in parallel across large ensembles of neurons, and at speeds that match the patterns of ongoing neural events. Optogenetics and reporters of neural activity already enable all-optical read-write operations to be performed on intact neural circuits without the need to implement electrodes within the brain circuits under study. To enable effective and efficient optical modulation and interrogation of neural circuits, optogenetic tools and reporters of neural activity must be implemented alongside tailored optical instruments that fully leverage their potential. To succeed, optical interfaces to the brain must be able to sculpt light at the same scale, resolution, and speed as individual neurons. Several light sculpting techniques have been developed to target individual neurons with light. Many of these techniques only satisfy some, but not all of the requirements listed above, thus limiting their practical applications. To achieve a leap forward in experimental capabilities, future optical approaches must be developed with a comprehensive design strategy that satisfies all the requirements dictated by the normal function of neurons for simultaneous and efficient brain interfacing. CGH is currently recognized as the most promising pathway to achieve this goal, as it enables the synthesis of custom illumination patterns that can simultaneously illuminate many individual neurons in parallel and with millisecond precision. To modulate neurons at the speed of neural events and manipulate ensembles of functionally defined neuron populations, CGH approaches must be driven by fast algorithms and yield spatially precise illumination patterns that will best achieve the desired coherent brain pattern. In our review, we highlighted the recent state of the art in CGH algorithms and how deep learning-based methods such as DeepCGH have the capacity to overcome both of these spatial and temporal challenges.

Deep learning-based CGH techniques consist of convolutional networks that are implemented on GPU-accelerated computers to achieve unprecedented speeds. The models are trained with thousands of example illumination patterns offline and do not require iterations to find solutions when the model is fully trained. Deep learning models also facilitate the development of task-optimized CGH applications by training CNNs to identify holograms that will best achieve the desired biological outcomes. Specialized training can be achieved by training CNN models with representative data sets and by tailoring the loss function to account for the biological response of individual neurons. Despite progress, existing CGH systems are fundamentally limited by their reliance on coherent light sources. The patterns they can synthesize must be obtained by shaping the 2D wavefront of a coherent wave, and the resulting volumetric illumination typically contains significant amounts of speckle noise. Even with the most advanced CGH hardware and algorithms that can identify the most suitable approximation of the desired illumination pattern, the mismatch between the requested, and rendered illumination patterns may be quite prohibitively large. As a result, 3D holograms often stimulate additional neurons beyond the ensembles being targeted even with optimized CGH hardware and algorithms. The next leap forward is to explore new light sculpting approaches that will be able to synthesize new illumination patterns that traditional CGH methods cannot achieve.

Advanced CGH techniques, designed not merely to produce high-quality images, but instead tailored to best respond to experimental needs are critical to extend the optogenetic capabilities of current CGH techniques. 3D MAP, e.g., leverages the specificity of optogenetic stimulation with an illumination strategy that enhances depth specificity to pinpoint neurons precisely in 3D and offers the best trade-off between accessible volume and 3D spatial resolution for neural targets that are sparsely distributed across brain tissue. Similarly, DeepCGH exploits both the speed and spatial modulation capabilities of DMDs to address neurons with multiple rapidly interleaving frames. Since DMDs operate far faster than the response time of bacterial opsins, individually coherent CGH frames displayed in a rapid sequence average together to create, from the perspective of the opsin, a perceptually incoherent illumination pattern that could not be obtained with conventional CGH techniques. Future developments of high-performance neural interfacing technologies will similarly need to account for experimental constraints across disciplines. We anticipate that the next generation of optical brain-machine interfaces will be developed through the joint design of high-performance opsins, more precise light sculpting techniques, and integrated CGH algorithms. Significant under-explored research opportunities exist at this intersection, in particular with ongoing efforts to achieving these same goals with miniature devices that are compatible with behavioral experiments that require unrestrained freely moving animals.

Last, it is important to highlight that innovative light sculpting techniques developed for neural interfacing applications have many other applications beyond the field of neuroscience. We anticipate that the ongoing efforts to develop optical interfaces for the brain will also have vital applications in biology and medicine where focusing light on specific cells deep into tissue also enables transformative experimental capabilities. Upgrading experimental systems with new CGH software is inexpensive and far less complicated than upgrading hardware. Hence, sharing new CGH algorithms with the broader community of neuroscientists can have a major impact on future progress in neuroscience and beyond. We, therefore, encourage our readers to be part of this effort by sharing their contributions to software and hardware development via open-access repositories accessible to all.

## Code, Data, and Materials Availability

The majority of Computer Generated Holography algorithms discussed in this review have been made available by their original authors on public repositories. Iterative exploration and iterative optimization CGH algorithms are available at https://github.com/UNC-optics/NOVO-CGH, along with a MATLAB script to compare CGH methods.^34^ The source code for DeepCGH^37^ is available at https://github.com/UNC-optics/DeepCGH, along with a hands-on tutorial available both on Google Colab and as a Jupyter Notebook. The source code for time-multiplexed dynamic CGH with iterative optimization^50^ is available at https://github.com/UNC-optics/DCGH.
