# Supplementary material for: Advances in computer-generated holography for targeted neuronal modulation
Source: Neurophotonics. 2022 Jun 16;9(4):041409. doi: 10.1117/1.NPh.9.4.041409 (PMC9201973; doi:10.1117/1.NPh.9.4.041409)
Supplement: Supplementary file 1 [file NPh_009_041409_SD001.pdf]

Supplementary material for "Advances in Computer Generated Holography for Targeted Neuronal Modulatiom"

M. Hossein Eybposh[a,b], **Vincent R. Curtis**[a,c], **Jose Rodriguez-Romaguera**[c,d,e,f], **Nicolas C. Pégard**[a,b,d,f,*]

[a]University of North Carolina at Chapel Hill, Department of Applied Physical Sciences, Chapel Hill, USA, NC 27599
[b]University of North Carolina at Chapel Hill, Department of Biomedical Engineering, Chapel Hill, USA, NC 27599
[c]Department of Psychiatry, University of North Carolina, Chapel Hill, USA, NC 27599
[d]Neuroscience Center, University of North Carolina, Chapel Hill, USA, NC 27599
[e]Carolina Institute for Developmental Disabilities, University of North Carolina, Chapel Hill, USA, NC 27599
[f]Carolina Stress Initiative, University of North Carolina, Chapel Hill, USA, NC 27599

Nomenclature

λ	Wavelength of the laser in the CGH set up
$\phi_{SLM}(x, y)$	Phase of the modulation at the SLM plane
$A_{Laser}(x, y)$	Spatial distribution of the laser intensity at the SLM plane
$A_{SLM}(x, y)$	Amplitude of the modulation at the SLM plane
f	focal length of the Fourier lens
h	Length of the SLM short axis
$I'(x, y, z)$	Rendered illumination pattern intensity at the image plane
$I(x, y, z)$	Target illumination pattern intensity at the image plane
I_0	Total intensity of the light at the image plane.
L	Span of the rendered illumination pattern along the x and y dimensions
m	number pixels/discretization along an axis
$M(x, y)$	Complex field representation of modulation at the SLM plane
M_X	Magnification factor in an optical relay
n	number frames in a time-multiplexed CGH
$P(x, y, z)$	3D Complex field in the image plane
$P_{SLM}(x, y)$	Complex field at the SLM plane
ps	pixel pitch of the light modulator
Z	Span of the rendered illumination pattern along the z dimension

1 On the importance of phase information in images and holography

Phase modulation CGH, specifically in a Fourier holography setup, is preferred to amplitude modulation. Linear modulators have more modulation capabilities than binary modulators, and phase modulators do not discard photons, as opposed to amplitude modulators. Also, the Fourier domain carries the majority of the information that is recognizable in visual images. A common way to illustrate how phase carries more information about an illumination distribution than amplitude is shown in Fig. S1. Here we take the Fourier transform of two images (Fig. S1a), then, we swap phase but keep the amplitude in the Fourier domain. Finally, we take the inverse Fourier transform of the new complex fields and display the resulting images (Fig. S1b). We observe that the majority of the visual information is transferred to the other image, following the phase swap. This shows that phase in the Fourier domain carries most of the relevant information about the illumination distribution. In a CGH setup, the phase of the modulator is analogous to the phase of the target image $I(x, y, z)$ in the Fourier domain since $I(x, y, z)$ and $M(x, y)$ are related through an optical Fourier transform.

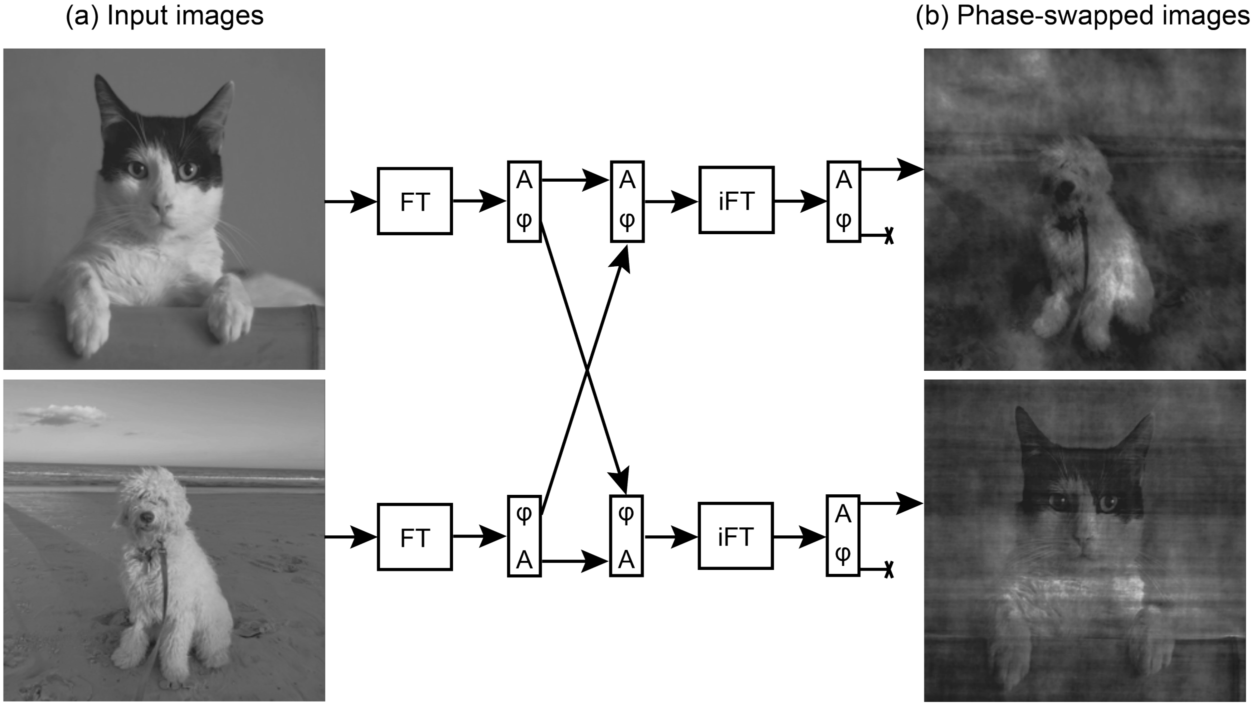

Fig 1 Phase in the Fourier domain carries most of the information about an intensity distribution in the image plane. (a) We swap the phase of two distinct images in the Fourier domain while keeping their respective amplitudes. We then compute the inverse Fourier transform (iFT) and display the resulting intensity of the reconstructed complex field. (b) Results show that the visual information of the image is mostly following the phase in the Fourier domain.

2 Definition of Accuracy

To evaluate the performance of CGH algorithms full reference image quality assessment metrics are often used. Here we use accuracy, because it is scale invariant, intuitive, and differentiable. Differentiability is a necessary condition for gradient descent based optimization of modulation patterns in algorithms that are based on direct optimization (e.g. NOVO-CGH) or deep learning

(e.g. DeepCGH). The accuracy, AC, is a measure of the similarity between the target illumination pattern, $I = |P(x, y, z)|^2$, and the rendered illumination pattern, $I' = |A'(x, y, z)|^2$). The accuracy is defined as:

$$AC(I, I') = \frac{\sum_{x,y,z} (I.I')}{\sqrt{\left[\sum_{x,y,z} I^2\right]\left[\sum_{x,y,z} I'^2\right]}}. \tag{1}$$

We note that $AC = 1$ when the target and reconstruction are identical after normalization, i.e. when $I = K\hat{I}$, with $K > 0$, and $0 \leq AC < 1$ when the distributions are mismatched.

3 Implementing DeepCGH, a brief tutorial

DeepCGH is implemented in Python programming language with Tensorflow deep learning framework. Deep learning models benefit from the architecture of GPUs for improved computation time. Hence, we recommend using DeepCGH on a GPU-accelerated computer. A complete tutorial of DeepCGH with a representative example and a pre-trained model is available online on the GitHub repository associated with the software package. The software package is tailored for optogenetic applications with a moderate size CNN model that achieves high-speed inference capability. The package includes two modules that work together: `DeepCGH` and `DeepCGH_Datasets`. First, the `DeepCGH_Datasets` module is used to synthesize data that is later used to train the `DeepCGH` model.

3.1 Parameters of `DeepCGH_Datasets`

The parameters of the `DeepCGH_Datasets` module are determined within a Python dictionary. For optogenetic applications the preferred data type for training DeepCGH is images that consist of randomly located disks matched to the dimensions of a neuron's soma. The intensity of disks is also assigned randomly to account for the expected variability in opsin expression. In practice, the intensity of each neuron can be easily adjusted to account for the spatially dependent diffraction efficiency of the experimental device by enhancing the brightness in targets located in the periphery of the accessible volume. Important parameters for the `DeepCGH_Datasets` module also include: the pixel size of the modulator, the number of planes segmenting the 3D volume, the number of randomly placed disks in each plane, the range of the randomly assigned disk intensities, the size of the disks, and the number of samples to be generated to create a training dataset. While the spatial resolution is determined by the available light modulator, the number of planes and disks within each plane are determined by the user based on experimental requirements. The number of samples needed for training depends on the capacity of the network and the number of parameters of the CNN. As a rule of thumb, the number of samples should be no less than 5,000 samples for very small models.

3.2 Parameters of `DeepCGH`

The parameters of the `DeepCGH` module are also stored within a Python dictionary. The physical distance between different depth planes is determined with the `plane_distance` parameter. The user can specify the capacity of the model by adjusting the number of kernels. Experiments indicate that depending on the difficulty of the learning task for the CNN, a wide range of kernels might be suitable. The difficulty of the learning task is dependent on the wavelength of the laser, the pixel

pitch of the SLM, and the focal length of the Fourier lens. We encourage attempting to generate DeepCGH models by beginning with a smaller number of kernels and gradually increasing it if the model performance is not satisfactory. The interleaving factor should remain as small as possible since it dramatically affects the speed of the CNN. Additional details that pertain on the advantages of interleaving, the selection of the interleaving factor, and its effect on computation speed and accuracy are available in[39]. Finally, our experiments have shown that beginning with small values for the learning rate and the batch size are helpful to ensure that new custom DeepCGH models can be trained.

3.3 Interleaving

The interleave module reduces the spatial dimensions of a tensor without losing information, simply by rearranging the pixels of the original input into multi-channel images of smaller size. The de-interleave module performs the inverse operation. This operation is demonstrated in Fig. S2 for an example interleaving factor of 2. The interleaving factor determines the spacing between pixels that are going to be rearranged next to each other.

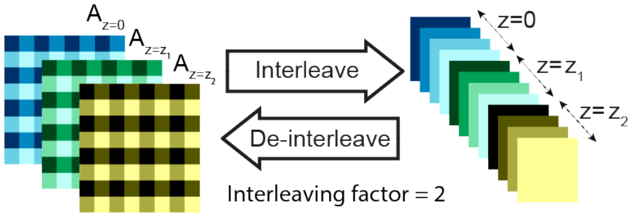

Fig 2 An interleaving operator systematically rearranges the pixels of the original input to create new images with smaller spatial dimension (along x and y axes) but with more channels. In this example the input is three gray-scale images representing the target illumination patterns at 3 different depths, $A_{z=0}$, $A_{z=z_1}$, and $A_{z=z_2}$. For an interleaving factor (IF) of 2, every two pixels along the spatial dimensions are assigned to the same channel. This results in 4 channels (IF^2) for each depth plane. Interleaving is an information-preserving operation, and De-interleaving represents the reverse operation.
